# Supplementary figures and images for: Regeneration-associated cells improve recovery from myocardial infarction through enhanced vasculogenesis, anti-inflammation, and cardiomyogenesis
Source: PLoS One. 2018 Nov 28;13(11):e0203244. doi: 10.1371/journal.pone.0203244 (PMC6261405; doi:10.1371/journal.pone.0203244)

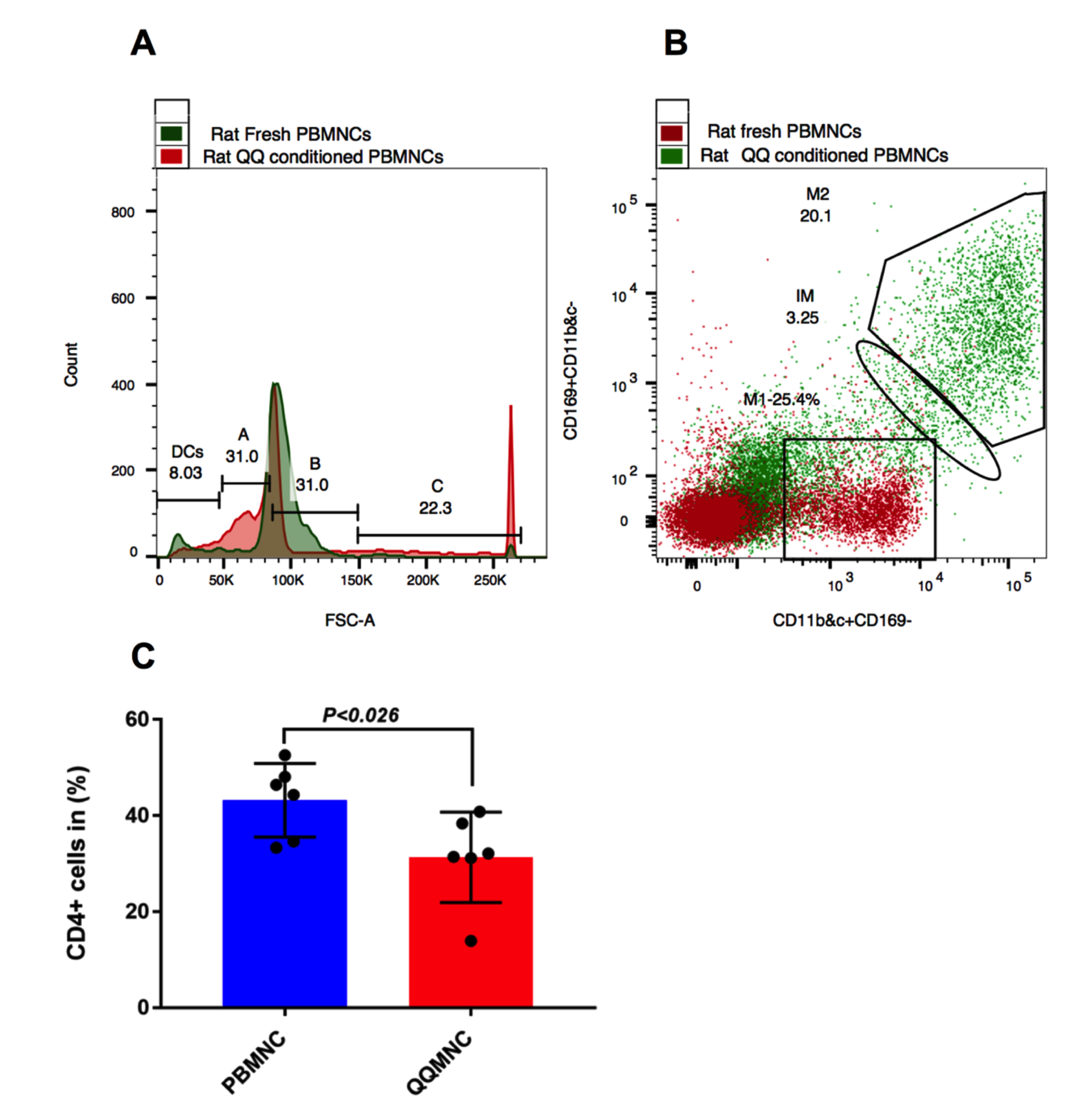

Supplement: S1 Fig — (A) In flow cytometry, QQMNCs, lymphocyte-sized cells A), monocyte-sized cells B) and macrophage-size cells populations C) were separately gated from total live cells and compared with freshly isolated PBMNCs. (B) As shown multicolor overlaid flow cytometric analyzing method, the percentage of M1ɸ was grater in freshly isolated PBMNCs while in QQMNCs anti-inflammatory and regenerative macrophages type 2 (M2ɸ) was markedly increased. (C) Post-QQ conditioning the number of CD4+ cells were decreased. Statistical significance was determined using Manny-Whitney test. Results represented as mean ± SEM. (TIFF) [file pone.0203244.s001.tiff]

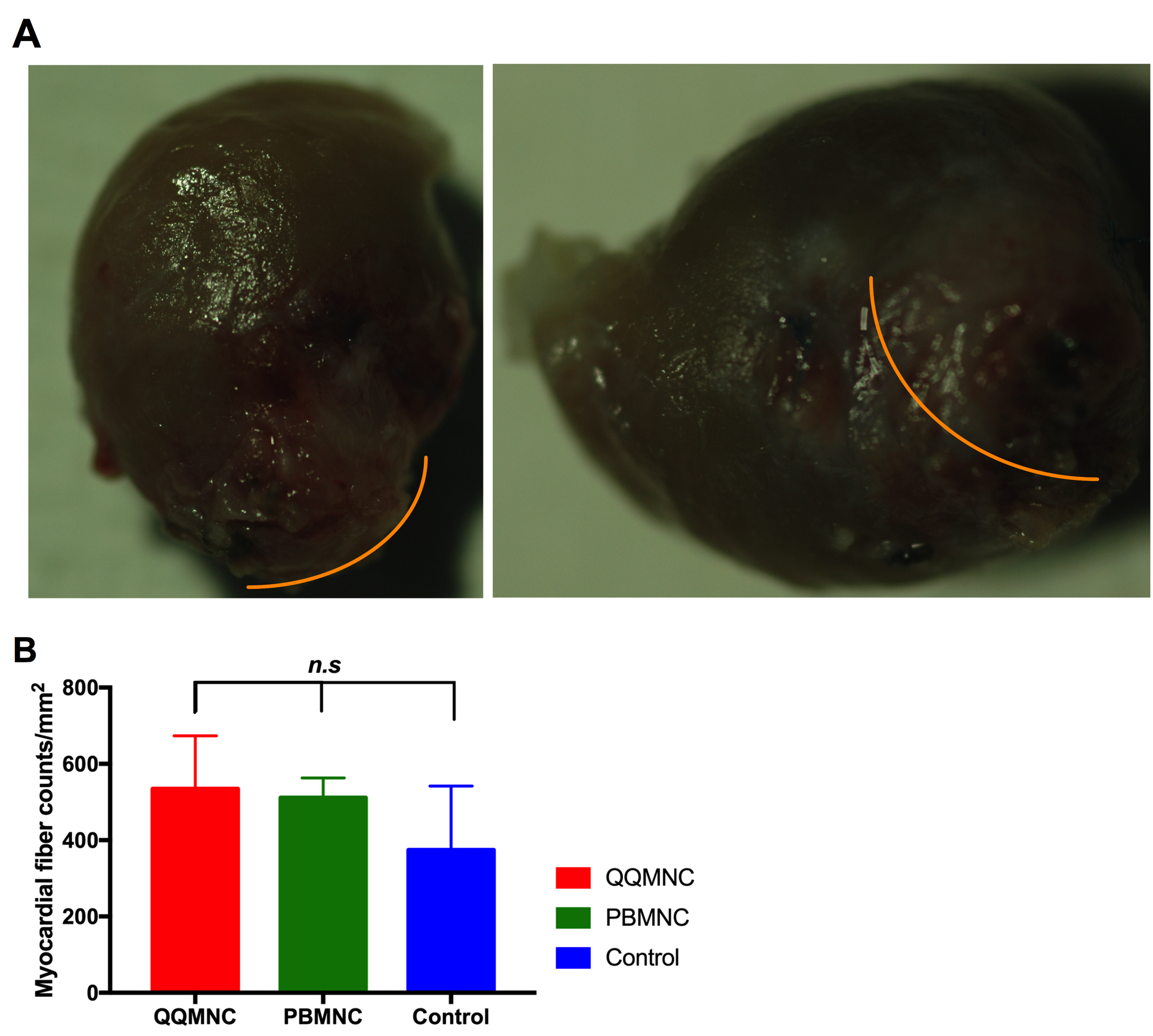

Supplement: S2 Fig — (A) PB-Tx and Control-Tx groups developed giant LV aneurysm 4 weeks later after MI induction. (B) Myocardial fiber counts at 4 weeks after myocardial infarction. (TIFF) [file pone.0203244.s002.tiff]
