# Supplementary material for: Regeneration-associated cells improve recovery from myocardial infarction through enhanced vasculogenesis, anti-inflammation, and cardiomyogenesis
Source: PLoS One. 2018 Nov 28;13(11):e0203244. doi: 10.1371/journal.pone.0203244 (PMC6261405; doi:10.1371/journal.pone.0203244)
Supplement: S1 Table — (DOCX) [file pone.0203244.s007.docx]

**S1 Table: QQ culture contend and used items.**

| **Items name** | **Company catalog No.** | **Final concentration** |
| --- | --- | --- |
| Stem line II Hematopoietic Stem Cell Expansion Medium | Sigma Aldrich,  S0192 |  |
| 6-well Primaria plate | BD Biociences,  353846 |  |
| Rat rh Stem cell factor | Peprotec,  AF-400-22 | 100 ng\ml |
| Rat rh Vascular endothelial growth factor | Peprotec,  400-31 | 50 ng\ml |
| Rat rh Trombopoetin | Peprotec,  AF-400-34 | 20 ng\ml |
| Rat rh Interleukin-6 | Peprotec,  400-06 | 20 ng\ml |
| Murine rh Flt-3 ligand | Peprotec,  300-19 | 100 ng\ml |
