# Supplementary material for: Regeneration-associated cells improve recovery from myocardial infarction through enhanced vasculogenesis, anti-inflammation, and cardiomyogenesis
Source: PLoS One. 2018 Nov 28;13(11):e0203244. doi: 10.1371/journal.pone.0203244 (PMC6261405; doi:10.1371/journal.pone.0203244)
Supplement: S2 Table — (DOCX) [file pone.0203244.s008.docx]

**S2 Table: Fluorophore-labeled monoclonal antibodies.**

| **Antibody** | **Isotype** | **Manufacture name & catalog #** |
| --- | --- | --- |
| CD3-FITC | FITC mouse IgM | BioLegent, #201403 and #401606 |
| CD4-PE/Cy7 | PE/Cy7 mouse IgG1, ƙ | BioLegent, #201516 and # 400126 |
| CD8a-AlexaFlour 647 | AlexaFlour 647 mouse IgG1 | BioLegent, #201710 and #400130 |
| CD25-PE | PE mouse IgG1, ƙ | BioLegent, #202105 and #400112 |
| CD11b/c-PerCP/Cy5.5 | PerCP/Cy5.5 mouse IgG2a | BioLegent, #201820 and #400258 |
| CD68-FITC | FITC mouse IgG1 | BIO-RAD, #MCA1209F and MCA341F |
| CD163- AlexaFlour 647 | AlexaFlour 647 mouse IgG1 | BIO-RAD, #MCA342A647 and MCA1209A647 |
| CD34-PE | PE mouse IgG1, ƙ | Novus Biologicals, #NBP2-29455 and BioLegent, #400112 |
