# Supplementary material for: Regeneration-associated cells improve recovery from myocardial infarction through enhanced vasculogenesis, anti-inflammation, and cardiomyogenesis
Source: PLoS One. 2018 Nov 28;13(11):e0203244. doi: 10.1371/journal.pone.0203244 (PMC6261405; doi:10.1371/journal.pone.0203244)
Supplement: S3 Table — (DOCX) [file pone.0203244.s009.docx]

**S3 Table: Primers list for RT-qPCR.**

| **Gene Name** | **Gene ID** | **Accession #** | **Sequence** |
| --- | --- | --- | --- |
| Angpt1 | 89807 | NM_053546.1 | F: GCAACACCAAGTCCGAATG  R: TTTCTTTTTGGTCTGCATTCAC |
| Angpt2 | 89805 | NM_134454.1 | F: GACATGGAGGACAAGCACAG  R: TGGACACCAGCACCTGAAG |
| Arg1 | 29221 | NM_017134.3 | F: TGGGAAAAGCCAATGAACA  R: TGCTTCCAATTGCCATACTG |
| Cd38 | 25668 | NM_013127.1 | F: AAAATGTTCACCCTGGAGGA  R: CTCCAATGTGGGCAAGAGTC |
| Egr2 | 114090 | NM_053633.1 | F: CTACCCGGTGGAAGACCTC  R: TCAATGTTGATCATGCCATCTC |
| Foxp3 | 317382 | NM_001108250.1 | F: AGGAGCCGGGAGAGTTTCT  R: CTCCAGAGACTGCACCACTTC |
| Ifng | 25712 | NM_138880.2 | F: CCTGGAGTTTGTGAAGAACAACT  R: GGAAGTTGGGGTAGGAAGGA |
| Igf1 | 24482 | NM_052807.2 | F: TTTCAGCGCAGCTGATGT  R: ATCCCGAAGGACCCTTGT |
| Il6 | 24498 | NM_012589.2 | F: CCTGGAGTTTGTGAAGAACAACT  R: GGAAGTTGGGGTAGGAAGGA |
| Il10 | 25325 | NM_012854.2 | F: CAGATTCCTTACTGCAGGACTTTA  R: CAAATGCTCCTTGATTTCTGG |
| Kit | 64030 | NM_022264.1 | F: CGACAACCAAAGCAACATCA  R: TTGGACACCAGAAAGGTGTAAG |
| Mmp2 | 81686 | NM_031054.2 | F: CTCCCCCAAAACAGACAAAG  R: TGTCCTTCAGCACAAAGAGG |
| Mmp9 | 81687 | NM_031055.1 | F: GGTCGCTCGGATGGTTATC  R: AGTTGCCCCCAGTTACAGTG |
| Myc | 24577 | NM_012603.2 | F: GAATTTTTGTCTATTTGGGGACA  R: GCATCGTCGTGACTGTCG |
| Pecam1 | 29583 | NM_031591.1 | F: TCCTGAGGGTCAAGGTAATAGC  R: CTCCAGACTGTACATCGTTACCC |
| Ly6al (Sca1) | 362935 | NM_001128099.1 | F: TGCTATGATGTCCTGTGTGCT  R: ATGGGATCCCCAAGCAAT |
| Tnf | 24835 | NM_012675.3 | F: CGTAGCCCACGTCGTAGC  R: GGTTGTCTTTGAGATCCATGC |
| Tgfb1 | 59086 | NM_021578.2 | F: ATGGTGGACCGCAACAAC  R: ACAGCAATGGGGGTTCTG |
| Vegfb | 89811 | NM_053549.1 | F: GCAACACCAAGTCCGAATG  R: TTTCTTTTTGGTCTGCATTCAC |
| Nkx2-5 | 114109 | NM_053651.1 | F: GACAAAGCCGAGACAGACG  R: AGGTACCGCTGTTGCTTGAA |
| Gata4 | 54254 | NM_144730.1 | F: CGAGACACCCCAATCTCG  R: GTAGTGTCCTGTCCCATCTCG |
| Myh6 | 29556 | NM_017239.2 | F: AATTCGACAAGATCGAGGACA  R: TGACGGTGACACAGAAGAGG |
| Tbx5 | 304514 | NM_001009964.1 | F: AATTGAGAACAACCCCTTCG  R: CCTGGGAACCACAGGATACTC |
| Mef2c | 499497 | XM_006231731.2 | F: GCCCTGAGTCTGAGGACAAG  R: CAAAGTTGGGAGGTGGAACA |
| Il1b | 24494 | NM_031512.2 | F: CAGGAAGGCAGTGTCACTCA  R: TCCCACGAGTCACAGAGGA |
